# Parental and Teacher Autonomy Support in Developing Self-Regulation Skills

**DOI:** 10.3390/bs15121621

**Published:** 2025-11-25

**Authors:** Mustafa Özgenel, Süleyman Avcı

**Affiliations:** 1 Department of Educational Sciences, Faculty of Education, İstanbul Sabahattin Zaim University, 34303 İstanbul, Türkiye; 2 Department of Educational Sciences, Faculty of Education, Marmara University, 34722 Istanbul, Türkiye; suleyman.avci@marmara.edu.tr

**Keywords:** parental autonomy support, teacher autonomy support, self-regulation, homework completion, achievement

## Abstract

Homework is a key learning activity that promotes students’ self-regulation, motivation, and academic achievement. Previous studies highlight the importance of parental and teacher autonomy support in fostering these outcomes, but the mechanisms underlying these relationships require further investigation. This study investigates the effects of parental and teacher autonomy support on students’ self-regulation skills, mathematics homework completion, and academic achievement. Additionally, it examines whether gender moderates these relationships. The research was conducted with 530 middle school students from five public schools in Istanbul, covering 5th, 6th, and 7th grades. Data were collected on teachers’ and parents’ autonomy support in homework, students’ self-regulation strategies, homework behaviors, and academic performance. Analyses were performed using SPSS 25 and AMOS 25 software, employing structural equation modeling (SEM) with mediation paths, multi-group path analysis, and correlation tests. The results indicate that both parental and teacher autonomy support positively influence students’ use of self-regulation strategies, which in turn enhances homework completion and academic success. Self-regulation was found to mediate these relationships, confirming its crucial role in academic outcomes. However, gender did not significantly moderate these associations. This study advances the understanding of how parental and teacher autonomy support influence self-regulation, homework behavior, and academic achievement, contributing to the existing literature. By examining the mediating role of self-regulation and the moderating effect of gender, it provides in-depth insights into variations in homework engagement and academic outcomes. Findings highlight the importance of autonomy-supportive practices by parents and teachers to foster students’ independent study skills. Future studies could extend these findings by examining subject-specific differences and longitudinal effect.

## 1. Introduction

Homework serves not only as a practice tool but also as a means for teaching self-regulation skills such as time management. As outlined by Cooper (2015) and Epstein and Van Voorhis (2012), homework tasks are designed to teach skills related to practice, preparation, and participation. During the primary school years, due to intense parental control (Cooper et al., 2000), students do not need skills such as organising the environment, managing distractions, and using time effectively. In secondary school, due to the change in school structure, the difficulty of subjects, and the desire for freedom during adolescence, parents (Boonk et al., 2018; Hill & Tyson, 2009; Lee, 2009; Maltese et al., 2012) shift to a supportive approach rather than a participative and controlling role (Boonk et al., 2018). As parental control decreases, students begin to use their own management skills to complete their homework. Students with effective self-regulation and homework management skills are more successful in homework completion and academic progress (Estévez et al., 2018; Núñez et al., 2015a; Valle et al., 2019; J. Xu et al., 2015; Yang & Tu, 2020; Yang & Xu, 2015). Differences in the acquisition and use of self-regulation skills depend on many variables, the two most important of which are parental and teacher involvement (Corno & Xu, 2004; Núñez et al., 2015a; J. Xu et al., 2014; J. Xu & Wu, 2013).

In today’s digital age, the widespread use of online homework platforms and hybrid learning environments has significantly increased the need for students to manage their own learning processes (Adam et al., 2017; Sharma et al., 2024). Similar to homework (Corno & Xu, 2004), students in distance learning contexts often participate without direct supervision from an adult, which once again underscores the importance of self-regulation skills. Since the use of self-regulation strategies largely depends on students’ own willingness (Zimmerman, 2002), it is essential to identify the factors that motivate them to engage in these processes. The findings of this study will provide valuable insights for teachers and parents seeking to enhance students’ engagement in learning activities in technology-driven contexts, while also highlighting the critical role of autonomy support in this process.

Parents can be involved in students’ academic work in supportive, autonomous, or controlling roles (Dumont et al., 2014; Moroni et al., 2015; Trautwein et al., 2006). The results of previous studies show that supportive and autonomy-oriented parenting practices are positively related to self-regulated learning skills, while controlling parenting practices have a negative effect (Dong et al., 2024; Dumont et al., 2012; Gonida & Cortina, 2014; Hill & Tyson, 2009; Núñez et al., 2015a; Silinskas & Kikas, 2019). Similarly, autonomy support provided by teachers is positively related to self-regulation skills (J. Xu, 2016, 2024; J. Xu et al., 2017). Therefore, the relationship between autonomy support provided by parents and teachers in homework and self-regulation skills needs to be investigated in more detail. To address this gap, the present study focuses on Türkiye, a country with a collectivist cultural orientation, and investigates the effects of parental and teacher autonomy support on students’ self-regulation skills, homework completion, and academic achievement, while also exploring whether these relationships differ by gender.

### 1.1. Self-Regulation of Homework

The absence of teacher guidance in the homework process encourages students to manage their own application processes. Therefore, homework is an environment in which self-regulated learning is applied. Homework management refers to students’ use of self-regulated learning strategies in the homework process (J. Xu & Corno, 2003). Self-regulation is an ongoing process in which individuals manage their thoughts, feelings, behaviours, and environment to achieve academic goals (Ramdass & Zimmerman, 2011). Students’ effective use of self-regulation strategies (goal setting, time management, effective use of learning strategies, attention management, maintenance of motivation and stress management) makes their learning processes more active and constructive and increases their academic achievement (Boekaerts & Corno, 2005; Dent & Koenka, 2016; Dignath & Büttner, 2008). Self-regulation is realised through three components: motivational, cognitive and metacognitive. The motivational dimension shows the individual’s belief in his/her own abilities (self-efficacy, expectancy) and the value he/she places on the task in terms of achieving the learning goal. The cognitive dimension is the use of strategies necessary to complete the task. The metacognitive dimension is the student’s monitoring of his/her own development during the homework process and intervening when necessary. The strategies often used in this context are orienting, planning, performing, monitoring, evaluating and correcting (Ramdass & Zimmerman, 2011; Trautwein & Köller, 2003; Zimmerman, 2000, 2008). 

Students’ ability to control their own behaviour, emotions and thoughts, i.e., volitional control, is of great importance when doing homework. Volitional control is one of the self-regulation models used in education. This model helps learners to regulate their behaviour in a way that enables them to achieve goals previously set by another authority (Boekaerts & Corno, 2005). Volitional control is a structure that encourages students to focus on their goals and to act in accordance with these goals. Particularly in the homework process, students use the strategies of organising the environment, managing time, focusing attention, monitoring motivation and controlling emotions in order to achieve the goals set by the teacher (Corno, 2011). These strategies were defined in J. Xu and Corno’s (1998) study and developed in Xu’s subsequent studies to form the Homework Management Scale (HMS) for middle and high school students (J. Xu, 2008; J. Xu et al., 2015; Yang & Xu, 2015). This scale was developed to assess students’ homework management strategies. As noted in J. Xu and Corno’s (2003) study, homework management is addressed in five basic dimensions (1) organising the environment; (2) managing time; (3) focusing attention; (4) monitoring motivation; and (5) controlling emotions) and these dimensions include strategies to help students succeed in homework (J. Xu, 2010).

### 1.2. Parental and Teacher Autonomy Support

Self-Determination Theory (SDT) emphasizes the importance of fulfilling three basic psychological needs (autonomy, competence, and relatedness) in the learning process. Satisfying these needs enhances students’ intrinsic motivation and supports their willingness to engage in learning behaviors voluntarily (Deci & Ryan, 2012).

Parental autonomy support is manifested through respecting students’ perspectives, encouraging them to make their own decisions, providing opportunities for independent choice, avoiding the use of controlling language, and fostering an autonomous home environment (Deci & Ryan, 2012). Such support not only strengthens students’ intrinsic motivation but also facilitates the use of self-regulation skills. Homework represents one of the most common home-based forms of parental involvement (Epstein, 1987; Epstein & Sanders, 2002). When parents provide autonomy-supportive assistance in the homework process, they positively influence students’ motivation and academic achievement (Cunha et al., 2015; Núñez et al., 2015b; Wei et al., 2019; Wilder, 2014; J. Xu et al., 2017). In this context, two studies carried out with middle and high school students in Turkey revealed that parental involvement positively influenced students’ time management, homework completion behaviors, and academic achievement (Avcı et al., 2025a, 2025c).

Teacher autonomy support in homework occurs across three dimensions: design, feedback, and autonomy provision (Epstein & Van Voorhis, 2012; J. Xu, 2016; J. Xu et al., 2022). It includes offering students choices in the selection, preparation, and presentation of assignments, valuing their preferences, and encouraging them to express themselves (Feng et al., 2019; Hagger et al., 2015; Yang & Xu, 2019). Such practices increase students’ perceived homework effort (Feng et al., 2019; J. Xu, 2016; Yang & Xu, 2019), homework completion (Hagger et al., 2015; J. Xu, 2016; Yang & Xu, 2019), and academic achievement (Trautwein et al., 2009; J. Xu, 2016, 2025). A study conducted with middle school students in Turkey revealed that teacher involvement positively influenced self-regulation skills and homework completion (Avcı et al., 2025b).

In sum, both parental and teacher autonomy support play a critical role in promoting students’ willingness to engage in learning, fostering the effective use of self-regulation strategies, and enhancing academic outcomes (Admiraal et al., 2024; Núñez et al., 2015b; Ramdass & Zimmerman, 2011; Rosário et al., 2009, 2018).

### 1.3. Moderating Effect of Gender

Girls and boys have different experiences of parental support and use of self-regulation skills. Parents use controlling and intrusive behaviours when helping boys (Bhanot & Jovanovic, 2005; Carter & Wojtkiewicz, 2000; Dumont et al., 2012), whereas they tend to use supportive behaviours when helping girls (Dumont et al., 2012). Males give up easily when faced with challenging tasks and lose focus and interest, leading parents to use controlling homework involvement (Silinskas & Kikas, 2019). Parents talk more to their daughters about school and expect more success from them (Carter & Wojtkiewicz, 2000).

It is generally accepted that girls devote more time to academic activities and have higher levels of achievement (Duckworth & Seligman, 2006). In general, girls put more effort into homework than boys (Avcı et al., 2025a; Cooper et al., 2006; Hong & Aqui, 2004). Girls are also more likely to complete homework assignments (Patall et al., 2008; J. Xu, 2010). One of the factors that increases girls’ success is that they use more self-regulation strategies (Pajares, 2002; Zimmerman & Martinez-Pons, 1990). Girls demonstrate better homework management skills than boys (Martin, 2004; J. Xu, 2006; J. Xu & Corno, 2006). According to J. Xu (2006) and J. Xu and Corno (2006), girls have better time management skills. Compared to boys, girls make more effort to manage their workspace, monitor motivation and control negative emotions (J. Xu, 2010). 

### 1.4. Theoretical Model

**Figure 1:**
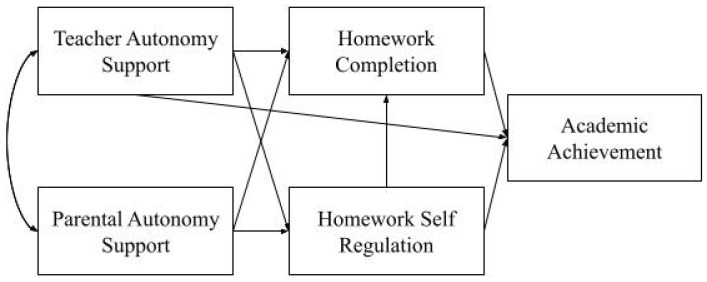
Theoretical model.

Autonomy support from parents and teachers in the context of homework is related to the use of self-regulation skills, homework completion and academic achievement. Pupils should be willing to use self-regulation skills in the homework process. Factors that motivate students to do homework also motivate them to use self-regulation skills. Research has shown that supportive parental involvement, including autonomy support, increases students’ self-regulated learning skills (Corno & Xu, 2004; W. Fan & Williams, 2010; Gonida & Cortina, 2014; Grijalva-Quiñonez et al., 2020; Luo et al., 2016; J. Xu & Wu, 2013). Research on autonomy support alone also supports the positive relationship between the two variables (J. Xu et al., 2015; J. Xu, 2016; J. Xu & Wu, 2013; Yang & Xu, 2015). Accordingly, it can be said that the autonomy support provided by parents to their children motivates students to use self-regulation skills. Similarly, the autonomy support provided by teachers to students in the context of homework is also related to self-regulation skills, as is the autonomy support provided by parents (J. Xu, 2016, 2024; J. Xu et al., 2017). In studies looking at teacher involvement in all its dimensions, the two variables were found to be positively correlated (J. Xu, 2023). The theoretical model based on these findings identified the pathways that explain the relationships between parental autonomy support, teacher autonomy support and self-efficacy (Figure 1). 

There is a large body of literature indicating that parental and teacher support for autonomy is positively associated with both homework completion and academic achievement. Research shows that, in general, teacher involvement (Ben-Eliyahu & Linnenbrink-Garcia, 2015; Cooper et al., 1998, 2006; Dettmers et al., 2010; Epstein & Van Voorhis, 2001; H. Fan et al., 2017; Fernández-Alonso et al., 2019; Flunger et al., 2017; Suárez et al., 2019; Trautwein et al., 2006) and specifically autonomy support (Hagger et al., 2015; J. Xu, 2016; Yang & Xu, 2019), homework completion is positively related to academic achievement. Teacher autonomy support is also positively related to academic achievement (Trautwein et al., 2009; J. Xu, 2016). Numerous studies have shown that parental involvement, including autonomy support, is positively related to homework completion and academic achievement (Doctoroff & Arnold, 2017; Gonida & Cortina, 2014; Moroni et al., 2015; Núñez et al., 2015b; Tam & Chan, 2009; Voorhis, 2011). Specifically, parental autonomy support increases students’ use of self-regulatory skills (W. Fan & Williams, 2010; Gonida & Cortina, 2014; Grijalva-Quiñonez et al., 2020; Luo et al., 2016). The theoretical model based on these findings identified pathways that explain the relationship between parental autonomy support, teacher autonomy support, homework completion and academic achievement (Figure 1). 

Evidence from homework-based research shows that homework management/self-regulation is positively related to a number of variables (Dettmers et al., 2009; Trautwein et al., 2006; J. Xu & Corno, 2022b). All components of homework management (arranging the environment, managing time, focusing attention, monitoring motivation and controlling emotions) affect homework completion (Estévez et al., 2018; J. Xu et al., 2015; Yang & Xu, 2015) and are positively related to academic performance (Núñez et al., 2015a; Valle et al., 2019; Yang & Tu, 2020). Similar results have been found in studies based on individual components of homework management. In particular, research on time management is at the forefront. Time management, homework completion (Rodríguez et al., 2019; Valle et al., 2016; J. Xu & Corno, 2022b), academic performance (J. Xu & Corno, 2022a) are positively related to self-regulation. In the theoretical model based on these findings, the pathways explaining the relationships between self-efficacy and homework completion and academic achievement were determined (Figure 1).

Trautwein et al. (2006) defined the mediating role between homework management/self-regulation skills, external variables (parental involvement, teacher involvement, student characteristics, etc.) and academic achievement in their homework model. In the model, self-regulation skills are related to homework completion and also positioned as a learning outcome. In this study, based on the literature showing the existence of positive dyadic relationships between variables and the model defined by Trautwein et al. (2006), the mediating role of self-regulation in the relationship between parental and teacher autonomy support and homework completion and academic achievement was determined (Figure 1). 

### 1.5. The Present Study

The primary purpose of this study is to examine the effects of parental and teacher autonomy support on students’ self-regulation skills, homework completion, and academic achievement. A secondary aim is to explore whether these relationships differ by gender. The theoretical basis of this study consists of self-determination theory, self-regulation theory and volition control theory. In the context of homework, parents’ and teachers’ support for self-determination is based on self-determination theory. The use of self-regulation skills in homework is referred to as homework management skills. Volitional control theory provides guidance for the use of self-regulation skills when goals are set. In homework, volitional control is used as the theoretical basis because the goals are set by the teachers (Boekaerts & Corno, 2005; Corno, 2011).

As the measurement tools in homework-based studies are domain-specific, the mathematics course was preferred in this study. In mathematics courses, more homework is assigned than in other subjects due to cultural reasons (Mu, 2014), national exams (Kitsantas et al., 2011), and the nature of the mathematics course (Holte, 2016; Kalsen et al., 2020; Kitsantas et al., 2011; Singh et al., 2013; Wu et al., 2022; J. Xu, 2015). In order to clearly determine the relationship between the variables, a course where homework is intensive should be preferred. Another reason for preferring mathematics is the study’s examination of parental autonomy support. In Turkiye, it is important to be successful in mathematics in order to be successful in national exams and to be able to study in prestigious schools. For this reason, parents appreciate and participate more in the mathematics course. The mathematics course provides a suitable environment to investigate parental involvement. Another reason is that there is a higher correlation between homework variables in mathematics compared to other subjects. (Cooper, 2015). At the same time, the impact of parental and teacher involvement on achievement is greater in mathematics homework than in other subjects (Wei et al., 2019; J. Xu & Corno, 2022b).

In line with the aims of the study, four research questions were identified: (1) Is there a significant relationship between parental and teacher autonomy support and students’ use of self-regulation strategies? (2) Is there a significant relationship between parental and teacher autonomy support and students’ homework completion and academic achievement? (3) Is there a significant relationship between students’ use of self-regulation strategies and students’ homework completion and academic achievement? (4) What is the mediating role of the use of self-regulation strategies in the relationship between parental and teacher autonomy support and students’ homework completion and academic achievement? (5) Do the relationships between parental and teacher autonomy support, use of self-regulation strategies, homework completion and academic achievement differ by gender? Hypotheses of the study:

**H1.**  *A positive relationship is expected between parental and teacher autonomy support and the use of self-regulation strategies (Boonk et al., 2018; M. Wang & Eccles, 2012; Q. Wang et al., 2007; Woolley & Bowen, 2007; J. Xu, 2016, 2024; J. Xu et al., 2018).*

**H2.**  *A positive relationship is expected between parental and teacher autonomy support and homework behavior as well as academic achievement (Doctoroff & Arnold, 2017; Gonida & Cortina, 2014; Hagger et al., 2015; Moroni et al., 2015; Tam & Chan, 2009; Yang & Xu, 2019)*.

**H3.**  *A positive relationship is expected between the use of self-regulation strategies and homework completion and academic achievement (Estévez et al., 2018; Núñez et al., 2015a; Valle et al., 2019; Yang & Tu, 2020; Yang & Xu, 2015; Z. Xu et al., 2023)*.

**H4.**  *A mediating role of self-regulation strategies is expected in the relationship be-tween parental and teacher autonomy support and homework completion as well as aca-demic achievement (Estévez et al., 2018; Núñez et al., 2015a; Valle et al., 2019; J. Xu et al., 2015; Yang & Tu, 2020; Yang & Xu, 2015)*.

**H5.**  *The relationships between parental and teacher autonomy support, the use of self-regulation strategies, homework completion, and academic achievement are expected to vary by gender (Bhanot & Jovanovic, 2005; Carter & Wojtkiewicz, 2000; Dumont et al., 2012; Pajares, 2002; Zimmerman & Martinez-Pons, 1990)*.

## 2. Materials and Methods

### 2.1. Participants and Implementation Process

Compulsory education in the Turkish education system lasts 12 years. The first four years are primary school, the second four years are middle school and the third four years are high school. This research was carried out on middle school students in the 5th, 6th and 7th grades. In Turkiye, at the end of the eighth grade, there is an exam for the transition from eighth grade subjects to high school. For this reason, students in the eighth grade can receive support from private tutors, free weekend courses at school or private tutoring centers. As a result, the factors influencing success in year 8, homework completion and parental attitudes may change. For these reasons, eighth graders were not included in this study. A total of 530 students, 56.2 percent female and 43.8 percent male, participated in the study. 28.5 percent of the students were in grade 5, 45.5 percent in grade 6 and 26 percent in grade 7. The mean age of the students was 11.63 years (SD = 0.93). Data were collected from 5 different public schools and 26 classrooms in Istanbul.

The research process was initiated by obtaining the necessary permissions from the Ministry of National Education and the relevant school administrations. Permission was also obtained from the teachers of the courses in which the study was to be conducted. Prior to the implementation phase of the research, written consent was obtained from the parents of the students. After these permissions and consents were obtained, the questionnaire forms were collected by the researchers during class time. In order not to influence the students’ opinions, class times when the mathematics teacher was not present were preferred. All implementations were successfully completed in March 2024.

### 2.2. Instruments

#### 2.2.1. Perceived Teacher Homework Autonomy Support

To determine teachers’ perceived autonomy support for homework, the Teacher Homework Involvement Scale developed by J. Xu (2016) and adapted into Turkish by Avcı and Özgenel (2024) was used. The scale consists of three sub-dimensions named as homework quality, feedback quality and autonomy support, 4 items in each sub-dimension and 12 items in total. Only the autonomy support dimension was used in this study. Autonomy support measures the extent to which teachers give students choices in the homework process and respect their choices (e.g., ‘My maths teacher listens to my ideas about homework’). The response scale is a 4-point Likert scale (1 = strongly disagree to 4 = strongly agree). The Cronbach’s alpha values of the Turkish version are 0.866, 0.848 and 0.863 for the dimensions of homework quality, feedback quality and autonomy support, respectively. The validation study (Avcı & Özgenel, 2024) reported satisfactory goodness-of-fit indices (X^2^/df = 1.599 < 3, CFI = 0.994 > 0.950, GFI = 0.994 > 0.950, TLI = 0.984 > 0.950, and RMSEA = 0.039 < 0.05), all of which meet recommended thresholds (Hu & Bentler, 1999; Kline, 2023).

#### 2.2.2. Perceived Parental Homework Autonomy Support

The Perceived Parental Homework Participation Scale, developed by J. Xu et al. (2017) and adapted into Turkish by Avcı and Özgenel (2024), was used to measure parents’ autonomy support in homework. The scale consists of two sub-dimensions: content-focused support (4 items) and autonomy-focused support (4 items). Autonomy-focused support involves parents listening to the child’s ideas about homework and expressing confidence in the child’s ability to do homework (e.g., ‘My parents listen to my ideas about maths homework’). The scale items are answered in a 4-point Likert format (1 = strongly disagree, 4 = strongly agree). The validation study (Avcı & Özgenel, 2024) reported satisfactory goodness-of-fit indices (χ^2^/df = 2.577, CFI = 0.973, GFI = 0.949, TLI = 0.964, RMSEA = 0.063), all of which meet the recommended thresholds (Hu & Bentler, 1999; Kline, 2023). Cronbach’s alpha internal reliability coefficients for content and autonomy were 0.858 and 0.809 respectively.

#### 2.2.3. Homework Self-Regulation

The Homework Management Scale developed by J. Xu (2008) and adapted into Turkish by Avcı and Özgenel (2024) was used to determine homework self-regulation strategies. The scale is Likert-type and consists of 22 items. The answers to the scale items are given on a scale expressed as (1) never, (2) rarely, (3) occasionally, (4) mostly, (5) always. There are five sub-dimensions in the scale: (a) organising the work environment (5 items) (e.g., “Find a quiet place”), (b) time management (4 items) (e.g., ‘Set priorities and plan ahead’), (c) dealing with distractions (5 items) (e.g., “Play around with other things while doing my mathematics homework”), (d) monitoring motivation (4 items) (e.g., “Praise myself for a good effort”) and (e) emotional control (e.g., “Tell myself to calm down”) (4 items). The confirmatory factor analysis reported in the previous study (Avcı & Özgenel, 2024) demonstrated satisfactory goodness-of-fit indices (χ^2^/df = 2.577, CFI = 0.973, GFI = 0.949, TLI = 0.964, RMSEA = 0.063), all of which met the recommended thresholds (Hu & Bentler, 1999; Kline, 2023). Cronbach’s alpha values for the subscales: 0.757 for organising the work environment, 0.786 for time management, 0.801 for dealing with distractions, 0.861 for motivation monitoring and 0.768 for emotional control. In this study, the total scores of the subdimensions were used in the analyses.

#### 2.2.4. Homework Completion

To determine students’ homework completion, two frequently preferred questions were used in the literature (J. Xu, 2011; J. Xu & Wu, 2013). The items are: (1) “How much of your assigned homework do you usually complete?” and (2) “How often do you come to class without your homework?” The response scale for the first item ranges from 1 (none) to 5 (all), while for the second item it ranges from 1 (never) to 5 (routinely). The Cronbach’s alpha internal reliability coefficient for the original scale is reported to be 0.71 (J. Xu, 2011). 

#### 2.2.5. Academic Achievement

Mathematics report card grades from the autumn semester (September 2023–January 2024) of the 2023–2024 academic year were used to measure students’ academic performance. The report card grade is a combination of two written exams and two performance grades.

### 2.3. Analysing Data

The data analysis process began with organising the data for analysis. In this study, the data of 8 students were excluded from the study due to the high probability of missing data. The rate of missing data for the remaining 530 students was determined to be 0.8% on average. To deal with the missing data in the database, the multiple imputation technique was used through the SPSS. Multiple imputation involves estimating missing values using observed data and adding random error to maintain variability in the data (Schafer & Graham, 2002). Box plots were then used to check for outliers. The results showed that there were no significant outliers (Kampstra, 2008). Skewness and kurtosis values were used to test the normality assumption of the data. The values obtained indicate that there is no significant deviation (<±2) from normality in the variables (Table 1). (Hair Jnr et al., 2010). Bivariate correlations between variables were assessed using Pearson’s correlation analysis. To determine whether indirect (mediated) relationships existed between the variables, analyses were conducted within the SEM framework using the bootstrapping method with 5000 resamples to estimate the 95% confidence intervals (CI) of the indirect effects. Mediation was considered confirmed if the bias-corrected 95% CI did not include zero. Control variables A and B were included in the mediation model. In addition, a multi-group path analysis was conducted to assess the comparability of the path model across different gender groups. The structural model was considered consistent if similar patterns were observed in multiple groups. When a tested structural model shows the same patterns across multiple groups, it indicates that the path coefficients, covariances, and error variances of the variables within the model are comparable (Jöreskog & Sörbom, 1993). If model invariance is not achieved, research can still provide valuable insights through group comparisons. Meredith (1993) proposes a four-stage logical process involving hypothesis testing methods to demonstrate invariance. The first stage is the configural model, where invariance at the configural level suggests that the model tested fits both groups. The second stage, metric invariance, involves testing the hypothesis that path coefficients are invariant across groups. The third stage, scalar invariance, tests the invariance of the intercepts of the path coefficients. Finally, strict invariance assesses the comparability of the error variances of the variables. In this study, these four models were sequentially compared using analysis of variance (ANOVA) to determine the degree of invariance, if any. In the second stage of model comparisons, the chi-square difference test was used to compare the goodness of fit of the models. This test helps to assess the difference in fit between two models. If the difference in chi-squared values between the two models is statistically significant, it indicates that the more restrictive model (e.g., metric, scalar or strict invariance model) has a worse fit. The results of the chi-squared difference test play an important role in determining whether the model is invariant between groups and the level of invariance achieved. IBM SPSS 25 and AMOS 25 software were used for the analyses.

## 3. Results

### 3.1. Preliminary Analysis

The research findings show that there are low and moderate positive relationships between teacher autonomy support, parental autonomy support, self-regulation strategy use, homework completion and academic achievement. First, a moderate positive relationship was found between teacher autonomy support (r = 0.436, *p* < 0.01) and parental autonomy support (r = 0.417, *p* < 0.01) and self-regulation strategy use. The relationship between teacher autonomy support (r = 0.259, *p* < 0.01) and parental autonomy support (r = 0.245, *p* < 0.01) and homework completion is quite similar. The relationship between teacher autonomy support (r = 0.240, *p* < 0.01) and academic achievement is higher than that between parental autonomy support (r = 0.122, *p* < 0.01). A moderate positive relationship was found between homework completion and academic achievement (r = 0.503, *p* < 0.01). 

These findings indicate that autonomy support, self-regulation, homework completion, and academic achievement are significantly and positively correlated, suggesting consistent preliminary relationships among the study variables.

A *t*-test was conducted to determine the interaction between gender and the variables in the study. According to the results, there was no difference between girls and boys in terms of teacher autonomy support, parental autonomy support, use of self-regulation strategies and homework completion scores (*p* > 0.05). Only academic achievement was partially higher for boys than for girls (*p* < 0.01). 

### 3.2. Structural Model

The results of the structural equation modeling (SEM) analysis, conducted to test the theoretical model and the hypothesized mediation paths (χ^2^/df (0.357/1) = 0.357 < 5, NFI = 0.99 > 0.95, CFI = 1.00 > 0.95, TLI = 1.00 > 0.90, RMSEA = 0.000 < 0.05) (Hu & Bentler, 1999; Kline, 2023). According to the SEM analysis, teacher autonomy support, parental autonomy support, use of self-regulation strategies and homework completion explained 27% of the variance in academic achievement. In addition, teacher autonomy support, parental autonomy support and use of self-regulation strategies explained 12% of the variance in homework completion. Teacher autonomy support and parental autonomy support explained 27% of the variance in self-regulation strategy use.

The results show that teacher autonomy support (β = 0.295) and parental autonomy support (β = 0.325) interact positively with the use of self-regulation strategies. It was found that students who received high levels of autonomy support from their parents and teachers used more self-regulation strategies in their homework. Teacher autonomy support (β = 0.135), parental autonomy support (β = 0.118), and self-regulation strategy use (β = 0.182) were positively related to homework completion. There was a strong interaction between homework completion and academic achievement (β = 0.488). Teacher autonomy support was positively related to academic achievement (β = 0.488), but the use of self-regulation strategies was negatively related (β = −0.083). Teacher autonomy support and parental autonomy support indirectly influence homework completion through the use of self-regulation strategies. Similarly, teacher autonomy support and parental autonomy support influence academic achievement through both self-regulation strategy use and homework completion behavior. Finally, the use of self-regulation strategies influences academic achievement through homework completion (Table 2).

The results show that parental and teacher autonomy support have significant positive effects on self-regulation, homework completion, and academic achievement, underscoring the importance of autonomy support in students’ learning processes.

### 3.3. Multi-Group Analysis by Gender

In the second stage of the study, a multi-group analysis was conducted to compare the effects of student gender on the relationships of the theoretical model. In order to analyse the invariance factor loadings, it was assumed that the factor loadings for the groups of girls and boys were not equal. The chi-squared value for the structural weights between the baseline model and the constraint model indicates that the structural parameter between the two groups was not statistically significant (CMIN = 5.205, *p* > 0.05). Accordingly, it can be said that gender is not a determinant variable in terms of the relationships in the theoretical model (Table 3). 

The multi-group analysis indicates that the structural relationships are largely consistent across genders, suggesting that the role of autonomy support does not substantially differ between male and female students.

## 4. Discussion

The results of this study highlight the effects of parental and teacher autonomy support on students’ self-regulation skills, homework completion, and academic achievement. Self-regulation, which includes organizing the environment, managing time, monitoring motivation, controlling emotions, and maintaining attention, was found to be positively associated with autonomy support. Furthermore, the study examined whether these relationships differed by gender, although no significant differences were observed.

### 4.1. Parental Autonomy Support, Teacher Autonomy Support and Self-Regulation

The results of the study support Hypothesis 1 (H1), which posits a positive relationship between parental and teacher autonomy support and the use of self-regulation strategies. The increase in parental and teacher autonomy support corresponds to an increase in self-regulation skills. The high level of interaction between autonomy support and self-regulation skills is consistent with the literature. Patall et al. (2008) emphasised that parental support is one of the factors that improve self-regulation. Secondary school students strive for freedom, especially as they enter adolescence (Eccles & Harold, 1993). In primary school, parents show more participatory parenting behaviour towards their children (Cooper et al., 2000). Parents check homework and help with homework. In adolescence, students do not demand this kind of parental involvement and resist pressure to do so. At the same time, as subjects become more difficult in secondary school, parents are no longer able to provide direct support (Patrikakou, 2004; Simon, 2001). In this process, students who are left alone to do homework begin to use their self-regulation skills more actively for success and find the opportunity to develop them. 

The positive interaction between parents’ and teachers’ autonomy support practices and self-regulation can be explained within the framework of self-determination theory. Autonomy support provided by teachers and parents directly interacts with autonomy, competence, and relatedness, which are the three basic psychological needs in self-determination theory. Teachers provide autonomy support by providing students with options that give them more control over their own learning processes. Providing autonomy also means believing in the learner, giving the learner opportunities to succeed, and constantly interacting with the learner. In this way, the needs for competence and relatedness are also met (Deci & Ryan, 2002). Psychological resources supported by these practices increase intrinsic motivation for homework (Assor et al., 2002; Reeve, 2009) It supports the use of self-regulation skills, which in turn leads to success. Intrinsic motivation increases when activities are aligned with their values and interests, and students are more likely to use self-regulation strategies (Ryan & Deci, 2000). 

The findings in this study that students use more self-regulation strategies as autonomy support increases are quite understandable and expected. Numerous studies show that there is a positive relationship between parental and teacher autonomy support and the use of self-regulation strategies (W. Fan & Williams, 2010; Gonida & Cortina, 2014; Grijalva-Quiñonez et al., 2020; Luo et al., 2016). For example, Grijalva-Quiñonez et al. (2020) found a low-level positive relationship between parental autonomy support and self-regulation in a study of students aged 9-12. Based on the findings, it is concluded that autonomy support from parents and teachers can positively affect students’ academic achievement by increasing their ability to manage their own learning processes. 

One of the distinctive aspects of this study is that it highlights the role of autonomy support in Türkiye, a country with a moderately collectivist cultural orientation (Komisarof & Akaliyski, 2025). Deci and Ryan (2012) emphasized that the need for autonomy and its positive effects are universal. In this regard, it can be argued that autonomy support provided by parents and teachers also produces positive outcomes in the specific academic context of homework, consistent with Self-Determination Theory (SDT). Similar findings were reported in J. Xu’s (2024) study conducted with Chinese students. Taken together, these results suggest that even within collectivist cultural contexts, autonomy support remains a strong predictor of students’ self-regulation skills, indicating that cultural differences do not diminish the strength of this relationship. This not only reinforces the universality of autonomy support but also underscores the importance of understanding how it operates across different cultural settings.

### 4.2. Mediator Role of Self-Regulation

The results of the study support the hypothesis (H2), which posits a positive relationship between parental and teacher autonomy support and homework behavior as well as academic achievement. Hypothesis 3 (H3), which proposed a positive relationship between the use of self-regulation strategies and both homework completion and academic achievement, was also supported. Furthermore, the hypothesis (H4), which emphasizes the mediating role of self-regulation strategies in the relationship between autonomy support and academic outcomes, was confirmed. These findings highlight the effects of autonomy support and self-regulation on student achievement. Autonomy support from both parents and teachers was positively related to homework completion directly and indirectly through the use of self-regulation strategies. The level at which autonomy support is most important and sought by students is secondary school. This is because students need parental support at all levels (Boonk et al., 2018). At the secondary school level, providing autonomy and a supportive learning environment is the most important type of support for positive academic outcomes (Doctoroff & Arnold, 2017; Dumont et al., 2012; Gonida & Cortina, 2014; Moroni et al., 2015; Núñez et al., 2015b; Pomerantz et al., 2007; Tam & Chan, 2009; Voorhis, 2011; Woolley & Bowen, 2007). At the same time, the stage at which self-regulation skills are most frequently used in the process of doing homework is secondary school. In primary school, self-regulation is newly acquired and there is not much need to use it, while in secondary school students do not prefer to use their strategies (Hong et al., 2009). Preferring to use self-regulation strategies to complete homework is an expected pattern of completion for secondary school students. Research findings also show the positive relationship between self-regulation and homework completion (Estévez et al., 2018; Valle et al., 2019; J. Xu et al., 2015; J. Xu, 2022; Yang & Xu, 2015). 

Support can also be drawn from self-regulation theory to explain the mediating role of self-regulation. Autonomy support motivates students to engage in relevant academic behaviours (Núñez et al., 2015a; Ramdass & Zimmerman, 2011; Rosário et al., 2009, 2018). With high intrinsic motivation, the student resorts to self-regulation strategies, which are also optional, in order to achieve academic results in the desired direction. It is the support of psychological resources through autonomy that internally mobilises the student (Ryan & Deci, 2000). In this study, different findings were obtained regarding the interaction between parental and teacher autonomy support and self-efficacy and academic achievement, as opposed to homework completion. First, parental autonomy support was not directly related to academic achievement but was found to be indirectly related through self-efficacy and homework completion. Teacher autonomy support was found to be related both directly and indirectly through self-efficacy and homework completion. The relationship between self-regulation and academic achievement is negative. Finally, and most importantly, there was a strong relationship between academic achievement and homework completion. There are some aspects of the research findings that are consistent and inconsistent with the general literature. The research findings on the positive relationship between supportive parental involvement and academic achievement predominate (Cooper et al., 2000; Dumont et al., 2012; Gonida & Cortina, 2014; Katz et al., 2011; Moroni et al., 2015; Núñez et al., 2015b; Tam & Chan, 2009; Voorhis, 2011). Accordingly, the results of this study are partially consistent with the literature. Due to the presence of many mediating variables between parental support and academic achievement, the relationship may have been indirect rather than direct. 

The results of the study showed that there was a positive relationship between teacher autonomy support and academic achievement in the expected direction. Although autonomy support was limited to homework, a similar positive relationship was found between academic achievement and homework completion. This suggests that teachers’ autonomy support is not specific to homework and that teachers with a certain style of completion may have similar attitudes in all classroom practices. The results obtained are in parallel with the studies conducted by Trautwein et al. (2009) and J. Xu (2016). In this study, while a positive relationship was observed between self-regulation and academic achievement, a negative relationship was found in the structural equation modelling (SEM) analysis. However, the effect of self-regulation on academic achievement through homework completion was found to be positive. It is generally reported in the literature that there is a positive relationship between these two variables (Valle et al., 2019; Yang & Tu, 2020). Therefore, it can be concluded that the results obtained are partially consistent with the literature.

### 4.3. Moderator Effect of Gender

The results of the study did not support Hypothesis 5 (H5), which proposed that gender moderates the relationships among the variables. The findings on the individual variables show that girls and boys are similar in terms of parental and teacher support for autonomy, self-regulation and homework completion. Only in mathematics were boys found to perform better than girls. The hypothesis that gender has a moderating effect in this study is based on the literature. According to the literature, girls do more homework (Patall et al., 2008; J. Xu, 2010) and use more self-regulation strategies (Pajares, 2002; Zimmerman & Martinez-Pons, 1990). In addition, families show more supportive participation towards girls (Carter & Wojtkiewicz, 2000; Dumont et al., 2012). The results of this study can be explained by the stereotypes that boys are better at mathematics. In Turkiye, girls perform better in the matriculation exam (LGS). According to the 2022 results, boys are sometimes more successful in mathematics, while girls are more successful in other subjects (science, social studies, Turkish, English, religion) (MoNE, 2022). According to the 2022 PISA report, girls and boys were equally successful in mathematics, while girls were more successful in reading (OECD, 2022). Therefore, the only explanation for girls’ underachievement in mathematics, all things being equal, is stereotyping. In this study, stereotyping may have played a role particularly in the use of self-regulation strategies and homework completion. 

### 4.4. Conclusions

The primary aim of this study was to investigate how parental and teacher autonomy support relate to students’ self-regulation, homework completion, and academic achievement. The findings demonstrate that self-regulation strategies play a mediating role in the link between autonomy support and academic outcomes, underscoring their central importance for student success. Moreover, the differentiated effects of parental and teacher autonomy support highlight the distinct yet complementary contributions of both contexts. Taken together, these results emphasize that fostering students’ self-regulation is a critical pathway to enhancing homework engagement and achievement.

### 4.5. Limitations and Future Research

This study has produced important findings that contribute to the homework literature. However, the study has several limitations. This study used a cross-sectional design, and the results only allow for relational inferences. Such a design makes it difficult to draw inferences about cause-effect relationships. As mentioned in the literature, cross-sectional studies collect data over limited periods of time, and this may not be sufficient to reflect the dynamics of interactions between variables over time. Another disadvantage of cross-sectional research is that the context of the survey and the characteristics of the respondents can significantly influence the results, and this cannot be controlled (Rindfleisch et al., 2008). Therefore, the results of this study are not sufficient to draw firm conclusions about the mechanisms underlying the observed relationships. The second limitation of the study is that it contains domain-specific results. As the measurement tools used in this study were domain-specific, the mathematics course was preferred. Therefore, the results reflect the mediating role of the use of self-regulation skills in relation to the mathematics course. At the same time, parents, teachers, homework completion and academic achievement also belong to the mathematics course. There may be differences in students’ completion in other subjects (Kitsantas et al., 2011). As emphasised in the findings on the mediating role of gender, the gender effect in mathematics course may be different in … course. Therefore, studies can be conducted to determine the interaction in different courses by using a similar research design. Another important limitation of the study that should be highlighted is related to the cultural context. The study produced results that reflect the characteristics of the Turkish culture. In addition, the research data was collected in a large metropolitan city in Turkiye and the participants were urban students. This is an important limitation in the process of generalising the findings. Parents living in large cities may generally have higher levels of education and income and may be more likely to adopt modern approaches to parenting. Therefore, the relevance of the findings to situations in rural areas may be limited and should be assessed taking into account the different socio-economic conditions in these areas. This suggests that the results of the study should be treated with caution when generalising to other cultural and geographical contexts.

The research findings have important implications for parents and teachers. Firstly, if parents adopt practices that support their children’s autonomy in homework support at secondary school level, this will help to improve their academic performance. In this context, it is recommended that they should be good listeners in order to get their children’s ideas. In addition, they should respect their children’s ideas and support them in putting their ideas into practice. An important part of promoting autonomy is trusting children and taking actions that demonstrate this trust. Having high academic expectations of children and emphasizing these will also increase motivation. Parents should support their children in learning and using self-regulation strategies such as time management, setting up a work environment, dealing with negative emotions and distractions. Parents should also avoid suppressive, punitive and controlling approaches to the homework process. The same suggestions listed for parents to support autonomy also apply to teachers. In addition, teachers should create homework options that take into account individual differences such as learning styles, learning levels and interests. In addition, students’ opinions should be taken into account when creating assignments. 

## Figures and Tables

**Table 1:** Descriptive Statistics of the participants and the Pearson correlations among the variables.

		1	2	3	4	5
1	Teachers Autonomy Support					
2	Parental Autonomy Support	0.376 **				
3	Homework Self-Regulation	0.436 **	0.417 **			
4	Homework Completion	0.259 **	0.245 **	0.291 **		
5	Academic Achievement	0.240 **	0.122 **	0.124 **	0.503 **	
	Skewness	−1.123	−0.476	−0.449	−0.924	−0.347
	Kurtosis	0.829	−0.474	−0.881	−0.121	0.693
	All M/Sd	3.07/0.89	3.45/1.21	3.47/0.64	4.17/0.92	74.99/16.51
	Girl M/Sd	3.12/0.86	3.52/1.13	3.51/0.57	4.21/0.88	72.87/16.43
	Boy M/Sd	3.01/0.92	3.37/1.30	3.42/0.72	4.13/0.97	77.72/16.23
	T test	1.345	1.452	1.469	0.924	−3.388 **

** *p* < 0.01.

**Table 2:** Standardised regression weights for middle student samples.

			Est.	CI_lower_	CI_upper_	*p*
***Direct Effect***						
Homework Self-Regulation	<---	Parental Autonomy Support	0.295	0.203	0.386	***
Homework Self-Regulation	<---	Teacher Autonomy Support	0.325	0.234	0.413	***
Homework Completion	<---	Teacher Autonomy Support	0.135	0.036	0.230	0.004
Homework Completion	<---	Homework Self-Regulation	0.182	0.078	0.284	***
Homework Completion	<---	Parental Autonomy Support	0.118	0.018	0.215	0.011
Achievement	<---	Homework Completion	0.488	0.409	0.562	***
Achievement	<---	Homework Self-Regulation	−0.083	−0.160	−0.009	0.048
Achievement	<---	Teacher Autonomy Support	0.150	0.070	0.235	***
***Indirect Effect***						
Homework Completion	<---	Teacher Autonomy Support	0.059	0.025	0.104	0.001
Homework Completion	<---	Parental Autonomy Support	0.054	0.025	0.090	0.001
Achievement	<---	Teacher Autonomy Support	0.068	0.014	0.122	0.014
Achievement	<---	Parental Autonomy Support	0.059	0.009	0.110	0.020
Achievement	<---	Homework Self-Regulation	0.089	0.039	0.144	0.002

*** *p* < 0.001.

**Table 3:** Multi-group analysis by gender.

	Df	$\chi^{2}$	$\chi^{2}$Δ	Df Δ	*p*
Unconstrained/Configural	2	2.283			
Structural weights/Scalar	8	5.205	2.922	6	0.735
Structural covariances/Strict	11	12.953	10.670	9	0.296

## Data Availability

The raw data supporting the conclusions of this article will be made available by the authors on request.

## References

[B1-behavsci-15-01621] Adam N. L., Alzahri F. B., Cik Soh S., Abu Bakar N., Mohamad Kamal N. A. (2017). Self-regulated learning and online learning: A systematic review. International Visual Informatics Conference.

[B2-behavsci-15-01621] Admiraal W., Lockhorst D., Post L., Kester L. (2024). Effects of students’ autonomy support on their self-regulated learning strategies: Three field experiments in secondary education. International Journal of Research in Education and Science (IJRES).

[B3-behavsci-15-01621] Assor A., Kaplan H., Roth G. (2002). Choice is good, but relevance is excellent: Autonomy-enhancing and suppressing teacher behaviours predicting students’ engagement in schoolwork. British Journal of Educational Psychology.

[B4-behavsci-15-01621] Avcı S., Özgenel M. (2024). Adaptation and psychometric evaluation of homework management, teacher and parent involvement scales for middle schoolers in Turkey. International Journal of Psychology and Educational Studies.

[B5-behavsci-15-01621] Avcı S., Özgenel M., Avcu A. (2025a). Gender and school level in relation to homework behavior, intrinsic motivation, and parental involvement. Psychology in the Schools.

[B6-behavsci-15-01621] Avcı S., Özgenel M., Avcu A. (2025b). Teacher involvement and self-regulation in homework: Impact on secondary school students’ homework behavior. Metacognition and Learning.

[B7-behavsci-15-01621] Avcı S., Özgenel M., Avcu A. (2025c). The importance of family participation in homework: Understanding the relationship between student homework behaviors and academic achievement by school level. Social Psychology of Education.

[B8-behavsci-15-01621] Ben-Eliyahu A., Linnenbrink-Garcia L. (2015). Integrating the regulation of affect, behaviour, and cognition into self-regulated learning paradigms among secondary and post-secondary students. Metacognition and Learning.

[B9-behavsci-15-01621] Bhanot R., Jovanovic J. (2005). Do parents’ academic gender stereotypes influence whether they intrude on their children’s homework?. Sex Roles.

[B10-behavsci-15-01621] Boekaerts M., Corno L. (2005). Self-regulation in the classroom: A perspective on assessment and intervention. Applied Psychology.

[B11-behavsci-15-01621] Boonk L., Gijselaers H. J. M., Ritzen H., Brand-Gruwel S. (2018). A review of the relationship between parental involvement indicators and academic achievement. Educational Research Review.

[B12-behavsci-15-01621] Carter R. S., Wojtkiewicz R. A. (2000). Parental involvement with adolescents’ education: Do daughters or sons get more help?. Adolescence.

[B13-behavsci-15-01621] Cooper H. (2015). The battle over homework: Common ground for administrators, teachers, and parents.

[B14-behavsci-15-01621] Cooper H., Lindsay J. J., Nye B. (2000). Homework in the home: How student, family, and parenting-style differences relate to the homework process. Contemporary Educational Psychology.

[B15-behavsci-15-01621] Cooper H., Lindsay J. J., Nye B., Greathouse S. (1998). Relationships among attitudes about homework, amount of homework assigned and completed, and student achievement. Journal of Educational Psychology.

[B16-behavsci-15-01621] Cooper H., Robinson J. C., Patall E. A. (2006). Does homework improve academic achievement? A synthesis of research, 1987–2003. Review of Educational Research.

[B17-behavsci-15-01621] Corno L. (2011). Studying self-regulation habits: Teachers college, columbia university. Handbook of self-regulation of learning and performance.

[B18-behavsci-15-01621] Corno L., Xu J. (2004). Homework as the job of childhood. Theory Into Practice.

[B19-behavsci-15-01621] Cunha J., Rosário P., Macedo L., Nunes A. R., Fuentes S., Pinto R., Suárez N. (2015). Parents’ conceptions of their homework involvement in elementary school. Psicothema.

[B20-behavsci-15-01621] Deci E. L., Ryan R. M. (2002). Self-determination research: Reflections and future directions.

[B21-behavsci-15-01621] Deci E. L., Ryan R. M. (2012). Self-determination theory. Handbook of Theories of Social Psychology.

[B22-behavsci-15-01621] Dent A. L., Koenka A. C. (2016). The relation between self-regulated learning and academic achievement across childhood and adolescence: A meta-analysis. Educational Psychology Review.

[B23-behavsci-15-01621] Dettmers S., Trautwein U., Lüdtke O. (2009). The relationship between homework time and achievement is not universal: Evidence from multilevel analyses in 40 countries. School Effectiveness and School Improvement.

[B24-behavsci-15-01621] Dettmers S., Trautwein U., Lüdtke O., Kunter M., Baumert J. (2010). Homework works if homework quality is high: Using multilevel modelling to predict the development of achievement in mathematics. Journal of Educational Psychology.

[B25-behavsci-15-01621] Dignath C., Büttner G. (2008). Components of fostering self-regulated learning among students. A meta-analysis on intervention studies at primary and secondary school level. Metacognition and Learning.

[B26-behavsci-15-01621] Doctoroff G. L., Arnold D. H. (2017). Doing homework together: The relation between parenting strategies, child engagement, and achievement. Journal of Applied Developmental Psychology.

[B27-behavsci-15-01621] Dong X., Yuan H., Xue H., Li Y., Jia L., Chen J., Shi Y., Zhang X. (2024). Factors influencing college students’ self-regulated learning in online learning environment: A systematic review. Nurse Education Today.

[B28-behavsci-15-01621] Duckworth A. L., Seligman M. E. (2006). Self-discipline gives girls the edge: Gender in self-discipline, grades, and achievement test scores. Journal of Educational Psychology.

[B29-behavsci-15-01621] Dumont H., Trautwein U., Lüdtke O., Neumann M., Niggli A., Schnyder I. (2012). Does parental homework involvement mediate the relationship between family background and educational outcomes?. Contemporary Educational Psychology.

[B30-behavsci-15-01621] Dumont H., Trautwein U., Nagy G., Nagengast B. (2014). Quality of parental homework involvement: Predictors and reciprocal relations with academic functioning in the reading domain. Journal of Educational Psychology.

[B31-behavsci-15-01621] Eccles J. S., Harold R. D. (1993). Parent-school involvement during the early adolescent years. Teachers College Record: The Voice of Scholarship in Education.

[B32-behavsci-15-01621] Epstein J. L. (1987). Parent involvement: What research says to administrators. Education and Urban Society.

[B33-behavsci-15-01621] Epstein J. L., Sanders M. G. (2002). Family, school, and community partnerships. Handbook of parenting: Vol. 5. Practical issues in parenting.

[B34-behavsci-15-01621] Epstein J. L., Van Voorhis F. L. (2001). More than minutes: Teachers’ roles in designing homework. Educational Psychologist.

[B35-behavsci-15-01621] Epstein J. L., Van Voorhis F. L. (2012). The changing debate: From assigning homework to designing homework. Contemporary debates in childhood education and development.

[B36-behavsci-15-01621] Estévez I., Regueiro B., Rodríguez S., Piñeiro I., Souto A., González-Sanmamed M. (2018). Why students of Secondary Education complete more homework?. European Journal of Investigation in Health, *Psychology and Education*.

[B37-behavsci-15-01621] Fan H., Xu J., Cai Z., He J., Fan X. (2017). Homework and students’ achievement in math and science: A 30-year meta-analysis, 1986–2015. Educational Research Review.

[B38-behavsci-15-01621] Fan W., Williams C. M. (2010). The effects of parental involvement on students’ academic self-efficacy, engagement and intrinsic motivation. Educational Psychology.

[B39-behavsci-15-01621] Feng X., Xie K., Gong S., Gao L., Cao Y. (2019). Effects of parental autonomy support and teacher support on middle school students’ homework effort: Homework autonomous motivation as mediator. Frontiers in Psychology.

[B40-behavsci-15-01621] Fernández-Alonso R., Woitschach P., Álvarez-Díaz M., González-López A. M., Cuesta M., Muñiz J. (2019). Homework and academic achievement in Latin America: A multilevel approach. Frontiers in Psychology.

[B41-behavsci-15-01621] Flunger B., Trautwein U., Nagengast B., Luedtke O., Niggli A., Schnyder I. (2017). A person-centered approach to homework behaviour: Students’ characteristics predict their homework learning type. Contemporary Educational Psychology.

[B42-behavsci-15-01621] Gonida E. N., Cortina K. S. (2014). Parental involvement in homework: Relations with parent and student achievement-related motivational beliefs and achievement. British Journal of Educational Psychology.

[B43-behavsci-15-01621] Grijalva-Quiñonez C. S., Valdés-Cuervo A. A., Parra-Pérez L. G., Vázquez F. I. G. (2020). Parental involvement in Mexican elementary students’ homework: Its relation with academic self-efficacy, self-regulated learning, and academic achievement. Psicología Educativa. Revista de Los Psicólogos de La Educación.

[B44-behavsci-15-01621] Hagger M. S., Sultan S., Hardcastle S. J., Chatzisarantis N. L. D. (2015). Perceived autonomy support and autonomous motivation towards mathematics activities in educational and out-of-school contexts is related to mathematics homework behaviour and attainment. Contemporary Educational Psychology.

[B45-behavsci-15-01621] Hair Jnr J. F., Black W. C., Babin B. J., Anderson R. E. (2010). Multivariate data analysis.

[B46-behavsci-15-01621] Hill N. E., Tyson D. F. (2009). Parental involvement in middle school: A meta-analytic assessment of the strategies that promote achievement. Developmental Psychology.

[B47-behavsci-15-01621] Holte K. L. (2016). Homework in primary school: Could it be made more child-friendly?. Studia Paedagogica.

[B48-behavsci-15-01621] Hong E., Aqui Y. (2004). Cognitive and motivational characteristics of adolescents gifted in mathematics: Comparisons among students with different types of giftedness. Gifted Child Quarterly.

[B49-behavsci-15-01621] Hong E., Peng Y., Rowell L. L. (2009). Homework self-regulation: Grade, gender, and achievement-level differences. Learning and Individual Differences.

[B50-behavsci-15-01621] Hu L. T., Bentler P. M. (1999). Cutoff criteria for fit indexes in covariance structure analysis: Conventional criteria versus new alternatives. Structural Equation Modeling: A Multidisciplinary Journal.

[B51-behavsci-15-01621] Jöreskog K. G., Sörbom D. (1993). LISREL 8: Structural equation modeling with the SIMPLIS command language.

[B52-behavsci-15-01621] Kalsen C., Kaplan İ., Şimşek M. (2020). Administrator, teacher and parent views on homework in primary schools. Abant İzzet Baysal University Journal of Faculty of Education.

[B53-behavsci-15-01621] Kampstra P. (2008). Beanplot: A boxplot alternative for visual comparison of distributions. Journal of Statistical Software.

[B54-behavsci-15-01621] Katz I., Kaplan A., Buzukashvily T. (2011). The role of parents’ motivation in students’ autonomous motivation for doing homework. Learning and Individual Differences.

[B55-behavsci-15-01621] Kitsantas A., Cheema J., Ware H. W. (2011). Mathematics achievement: The role of homework and self-efficacy beliefs. Journal of Advanced Academics.

[B56-behavsci-15-01621] Kline R. B. (2023). Principles and practice of structural equation modeling.

[B57-behavsci-15-01621] Komisarof A., Akaliyski P. (2025). New developments in Hofstede’s Individualism-Collectivism: A guide for scholars, educators, trainers, and other practitioners. International Journal of Intercultural Relations.

[B58-behavsci-15-01621] Lee J. (2009). Universals and specifics of math self-concept, math self-efficacy, and math anxiety across 41 PISA 2003 participating countries. Learning and Individual Differences.

[B59-behavsci-15-01621] Luo W., Ng P. T., Lee K., Aye K. M. (2016). Self-efficacy, value, and achievement emotions as mediators between parenting practice and homework behaviour: A control-value theory perspective. Learning and Individual Differences.

[B60-behavsci-15-01621] Maltese A. V., Tai R. H., Fan X. (2012). When is homework worth the time? Evaluating the association between homework and achievement in high school science and maths. The High School Journal.

[B61-behavsci-15-01621] Martin A. J. (2004). School motivation of boys and girls: Differences of degree, differences of kind, or both?. Australian Journal of Psychology.

[B62-behavsci-15-01621] Meredith W. (1993). Measurement invariance, factor analysis and factorial invariance. Psychometrika.

[B63-behavsci-15-01621] MoNE (2022). 20—2022 central examination report on secondary education institutions.

[B64-behavsci-15-01621] Moroni S., Dumont H., Trautwein U., Niggli A., Baeriswyl F. (2015). The need to distinguish between quantity and quality in research on parental involvement: The example of parental help with homework. The Journal of Educational Research.

[B65-behavsci-15-01621] Mu G. M. (2014). Chinese Australians’ Chineseness and their mathematics achievement: The role of habitus. The Australian Educational Researcher.

[B66-behavsci-15-01621] Núñez J. C., Suárez N., Rosário P., Vallejo G., Cerezo R., Valle A. (2015a). Teachers’ feedback on homework, homework-related behaviours, and academic achievement. The Journal of Educational Research.

[B67-behavsci-15-01621] Núñez J. C., Suárez N., Rosário P., Vallejo G., Valle A., Epstein J. L. (2015b). Relationships between perceived parental involvement in homework, student homework behaviours, and academic achievement: Differences among elementary, junior high, and high school students. Metacognition and Learning.

[B68-behavsci-15-01621] OECD (2022). PISA 2022 results.

[B69-behavsci-15-01621] Pajares F. (2002). Gender and perceived self-efficacy in self-regulated learning. Theory Into Practice.

[B70-behavsci-15-01621] Patall E. A., Cooper H., Robinson J. C. (2008). Parent involvement in homework: A research synthesis. Review of Educational Research.

[B71-behavsci-15-01621] Patrikakou E. (2004). Adolescence: Are parents relevant to students’ high school achievement and postsecondary attainment.

[B72-behavsci-15-01621] Pomerantz E. M., Moorman E. A., Litwack S. D. (2007). The how, whom, and why of parents’ involvement in children’s schooling: More is not necessarily better. Review of Educational Research.

[B73-behavsci-15-01621] Ramdass D., Zimmerman B. J. (2011). Developing self-regulation skills: The important role of homework. Journal of Advanced Academics.

[B74-behavsci-15-01621] Reeve J. (2009). Why teachers adopt a controlling motivating style towards students and how they can become more autonomy supportive. Educational Psychologist.

[B75-behavsci-15-01621] Rindfleisch A., Malter A. J., Ganesan S., Moorman C. (2008). Cross-sectional versus longitudinal survey research: Concepts, findings, and guidelines. Journal of Marketing Research.

[B76-behavsci-15-01621] Rodríguez S., Núñez J. C., Valle A., Freire C., del Mar Ferradás M., Rodríguez-Llorente C. (2019). Relationship between students’ prior academic achievement and homework behavioural engagement: The mediating/moderating role of learning motivation. Frontiers in Psychology.

[B77-behavsci-15-01621] Rosário P., Costa M., Núñez J. C., González-Pienda J., Solano P., Valle A. (2009). Academic procrastination: Associations with Personal, school, and family variables. The Spanish Journal of Psychology.

[B78-behavsci-15-01621] Rosário P., Núñez J. C., Vallejo G., Nunes T., Cunha J., Fuentes S., Valle A. (2018). Homework purposes, homework behaviours, and academic achievement. Examining the mediating role of students’ perceived homework quality. Contemporary Educational Psychology.

[B79-behavsci-15-01621] Ryan R. M., Deci E. L. (2000). Intrinsic and extrinsic motivations: Classic definitions and new directions. Contemporary Educational Psychology.

[B80-behavsci-15-01621] Schafer J. L., Graham J. W. (2002). Missing data: Our view of the state of the art. Psychological Methods.

[B81-behavsci-15-01621] Sharma K., Nguyen A., Hong Y. (2024). Self-regulation and shared regulation in collaborative learning in adaptive digital learning environments: A systematic review of empirical studies. British Journal of Educational Technology.

[B82-behavsci-15-01621] Silinskas G., Kikas E. (2019). Parental involvement in maths homework: Links to children’s performance and motivation. Scandinavian Journal of Educational Research.

[B83-behavsci-15-01621] Simon B. S. (2001). Family involvement in high school: Predictors and effects. NASSP Bulletin.

[B84-behavsci-15-01621] Singh P., Sidhu G. K., Fook C. Y. (2013). Malaysian parents’ practices and perspectives on the organisation of school homework. Pertanika Journal of Social Sciences & Humanities.

[B85-behavsci-15-01621] Suárez N., Regueiro B., Estévez I., del Mar Ferradás M., Guisande M. A., Rodríguez S. (2019). Individual precursors of student homework behavioural engagement: The role of intrinsic motivation, perceived homework utility and homework attitude. Frontiers in Psychology.

[B86-behavsci-15-01621] Tam V. C., Chan R. M. (2009). Parental involvement in primary children’s homework in Hong Kong. School Community Journal.

[B87-behavsci-15-01621] Trautwein U., Köller O. (2003). The relationship between homework and achievement-Still much of a mystery. Educational Psychology Review.

[B88-behavsci-15-01621] Trautwein U., Lüdtke O., Schnyder I., Niggli A. (2006). Predicting homework effort: Support for a domain-specific, multilevel homework model. Journal of Educational Psychology.

[B89-behavsci-15-01621] Trautwein U., Niggli A., Schnyder I., Lüdtke O. (2009). Between-teacher differences in homework assignments and the development of students’ homework effort, homework emotions, and achievement. Journal of Educational Psychology.

[B90-behavsci-15-01621] Valle A., Piñeiro I., Rodríguez S., Regueiro B., Freire C., Rosário P. (2019). Time spent and time management in homework in elementary school students: A person-centred approach. Psicothema.

[B91-behavsci-15-01621] Valle A., Regueiro B., Núñez J. C., Rodríguez S., Piñeiro I., Rosário P. (2016). Academic goals, student homework engagement, and academic achievement in elementary school. Frontiers in Psychology.

[B92-behavsci-15-01621] Voorhis F. L. V. (2011). Costs and benefits of family involvement in homework. Journal of Advanced Academics.

[B93-behavsci-15-01621] Wang M., Eccles J. S. (2012). Social support matters: Longitudinal effects of social support on three dimensions of school engagement from middle to high school. Child Development.

[B94-behavsci-15-01621] Wang Q., Pomerantz E. M., Chen H. (2007). The role of parents’ control in early adolescents’ psychological functioning: A longitudinal investigation in the United States and China. Child Development.

[B95-behavsci-15-01621] Wei J., Pomerantz E. M., Ng F. F.-Y., Yu Y., Wang M., Wang Q. (2019). Why does parents’ involvement in youth’s learning vary across elementary, middle, and high school?. Contemporary Educational Psychology.

[B96-behavsci-15-01621] Wilder S. (2014). Effects of parental involvement on academic achievement: A meta-synthesis. Educational Review.

[B97-behavsci-15-01621] Woolley M. E., Bowen G. L. (2007). In the context of risk: Supportive Adults and the school engagement of middle school students. Family Relations.

[B98-behavsci-15-01621] Wu J., Barger M. M., Oh D. (Diana), Pomerantz E. M. (2022). Parents’ daily involvement in children’s maths homework and activities during early elementary school. Child Development.

[B99-behavsci-15-01621] Xu J. (2006). Gender and homework management reported by high school students. Educational Psychology.

[B100-behavsci-15-01621] Xu J. (2008). Validation of scores on the homework management scale for high school students. Educational and Psychological Measurement.

[B101-behavsci-15-01621] Xu J. (2010). Gender and homework management reported by African American students. Educational Psychology.

[B102-behavsci-15-01621] Xu J. (2011). Homework completion at the secondary school level: A multilevel analysis. The Journal of Educational Research.

[B103-behavsci-15-01621] Xu J. (2015). Investigating factors that influence conventional distraction and tech-related distraction in maths homework. Computers & Education.

[B104-behavsci-15-01621] Xu J. (2016). A study of the validity and reliability of the Teacher Homework Involvement Scale: A psychometric evaluation. Measurement.

[B105-behavsci-15-01621] Xu J. (2022). More than minutes: A person-centred approach to homework time, homework time management, and homework procrastination. Contemporary Educational Psychology.

[B106-behavsci-15-01621] Xu J. (2023). Individual and class-level factors for students’ management of homework environment: The self-regulation perspective. Current Psychology.

[B107-behavsci-15-01621] Xu J. (2024). Homework time management: Do teacher and parent autonomy support matter?. Social Psychology of Education.

[B108-behavsci-15-01621] Xu J. (2025). Longitudinal relations among perceived autonomy support, time management, completion, and achievement. Studies in Educational Evaluation.

[B109-behavsci-15-01621] Xu J., Corno L. (1998). Case studies of families doing third-grade homework. Teachers College Record: The Voice of Scholarship in Education.

[B110-behavsci-15-01621] Xu J., Corno L. (2003). Family Help and Homework Management Reported by Middle School Students. The Elementary School Journal.

[B111-behavsci-15-01621] Xu J., Corno L. (2006). Gender, family help, and homework management reported by rural middle school students. Journal of Research in Rural Education.

[B112-behavsci-15-01621] Xu J., Corno L. (2022a). A person-centred approach to understanding self-regulation in homework using latent profile analysis. Educational Psychology.

[B113-behavsci-15-01621] Xu J., Corno L. (2022b). Extending a model of homework: A multilevel analysis with Chinese middle school students. Metacognition and Learning.

[B114-behavsci-15-01621] Xu J., Du J., Wu S., Ripple H., Cosgriff A. (2018). Reciprocal effects among parental homework support, effort, and achievement? An empirical investigation. Frontiers in Psychology.

[B115-behavsci-15-01621] Xu J., Fan X., Du J. (2015). Homework management scale: Confırmıng the factor structure wıth middle school students in China. Psychology in the Schools.

[B116-behavsci-15-01621] Xu J., Fan X., Du J., He M. (2017). A study of the validity and reliability of the parental homework support scale. Measurement.

[B117-behavsci-15-01621] Xu J., Wang C., Du J., Núñez J. C. (2022). Profiles of student-perceived teacher homework involvement, and their associations with homework behaviour and mathematics achievement: A person-centered approach. Learning and Individual Differences.

[B118-behavsci-15-01621] Xu J., Wu H. (2013). Self-regulation of homework behaviour: Homework management at the secondary school level. The Journal of Educational Research.

[B119-behavsci-15-01621] Xu J., Yuan R., Xu B., Xu M. (2014). Modelling students’ time management in math homework. Learning and Individual Differences.

[B120-behavsci-15-01621] Xu Z., Zhao Y., Liew J., Zhou X., Kogut A. (2023). Synthesizing research evidence on self-regulated learning and academic achievement in online and blended learning environments: A scoping review. Educational Research Review.

[B121-behavsci-15-01621] Yang F., Tu M. (2020). Self-regulation of homework behaviour: Relating grade, gender, and achievement to homework management. Educational Psychology.

[B122-behavsci-15-01621] Yang F., Xu J. (2015). Examining the psychometric properties of the homework management scale for high school students in China. Journal of Psychoeducational Assessment.

[B123-behavsci-15-01621] Yang F., Xu J. (2019). A psychometric evaluation of teacher homework involvement scale in online learning environments. Current Psychology.

[B124-behavsci-15-01621] Zimmerman B. J. (2000). Self-efficacy: An essential motive to learn. Contemporary Educational Psychology.

[B125-behavsci-15-01621] Zimmerman B. J. (2002). Becoming a self-regulated learner: An overview. Theory into Practice.

[B126-behavsci-15-01621] Zimmerman B. J. (2008). Investigating self-regulation and motivation: Historical background, methodological developments, and future prospects. American Educational Research Journal.

[B127-behavsci-15-01621] Zimmerman B. J., Martinez-Pons M. (1990). Student differences in self-regulated learning: Relating grade, sex, and giftedness to self-efficacy and strategy use. Journal of Educational Psychology.

